# Unveiling the Culprit: Sarcina Infection in the Stomach and Its Link to Unexplained Weight Loss

**DOI:** 10.7759/cureus.44565

**Published:** 2023-09-02

**Authors:** Richard Maradiaga, Shiab Mussad, Martha Yearsley, Subhankar Chakraborty

**Affiliations:** 1 Internal Medicine, The Ohio State University College of Medicine, Columbus, USA; 2 Pathology, The Ohio State University College of Medicine, Columbus, USA; 3 Gastroenterology, The Ohio State University College of Medicine, Columbus, USA

**Keywords:** diabetes, gastroparesis, impaired gastric motility, weight loss, sarcina species

## Abstract

*Sarcina* species (spp.) infections in humans are relatively rare; however, reported cases have recently increased. We have presented the case of a 56-year-old female with diabetes who presented with bloating, dysphagia, and substantial weight loss, ultimately diagnosed with reactive gastritis secondary to *Sarcina *spp. infection. Administration of antibiotics and a proton pump inhibitor led to symptom alleviation and weight gain. This case underscores the significance of considering *Sarcina *spp. infection in patients experiencing unexplained weight loss and nonspecific gastrointestinal symptoms, highlighting the importance of promptly identifying and managing these infections to prevent potentially life-threatening complications that are becoming more prevalent in literature.

## Introduction

Unintentional weight loss, characterized as an unexplained reduction exceeding 5% of body weight within six months, in conjunction with gastrointestinal symptoms, warrants consideration of underlying pathology [1]. Such weight loss is associated with a heightened risk of substantial morbidity and mortality, encompassing various non-malignant and malignant gastrointestinal disorders. These conditions include chronic inflammatory bowel diseases, malabsorption syndromes, functional gastrointestinal disorders (such as gastroparesis and irritable bowel syndrome), and gastrointestinal infections (including Helicobacter pylori and parasitic infections). These conditions disrupt normal nutrient digestion and absorption, leading to weight loss. A thorough investigation is imperative, as fewer common etiologies may be present. 

*Sarcina *spp. organisms, *Sarcina ventriculi *(SV) or *Clostridium ventriculi* (CV), are increasing in incidence. *Sarcina *spp. infections have been documented in the esophagus, stomach, and duodenum [2]. Weight loss, accompanied by abdominal pain, dysphagia, bloating, abdominal distention, and emesis, is frequently reported as presenting symptoms of Sarcina spp. infections. Case reports have detailed severe complications, including gastric outlet obstruction, emphysematous gastritis, ulcerations, gastrointestinal bleeding, visceral perforation, and underlying malignancy [2,3]. Furthermore, no consensus has been reached regarding its optimal management. Here, we present the case of a 56-year-old female with uncontrolled diabetes who experienced bloating, dysphagia, and significant weight loss, ultimately found to have reactive gastritis secondary to *Sarcina* spp. infection.

## Case presentation

A 56-year-old female patient presented with progressively worsening symptoms, including bloating, reflux, intermittent dysphagia, emesis, and regurgitation, alongside a substantial weight loss of over 100 lbs. within two years (from a baseline weight of 250 lbs. to 141 lbs.). The patient's medical history was notable for diastolic heart failure, ongoing tobacco use, depression, hypothyroidism, and uncontrolled type 2 diabetes mellitus complicated by a diabetic foot ulcer.

**Figure 1:**
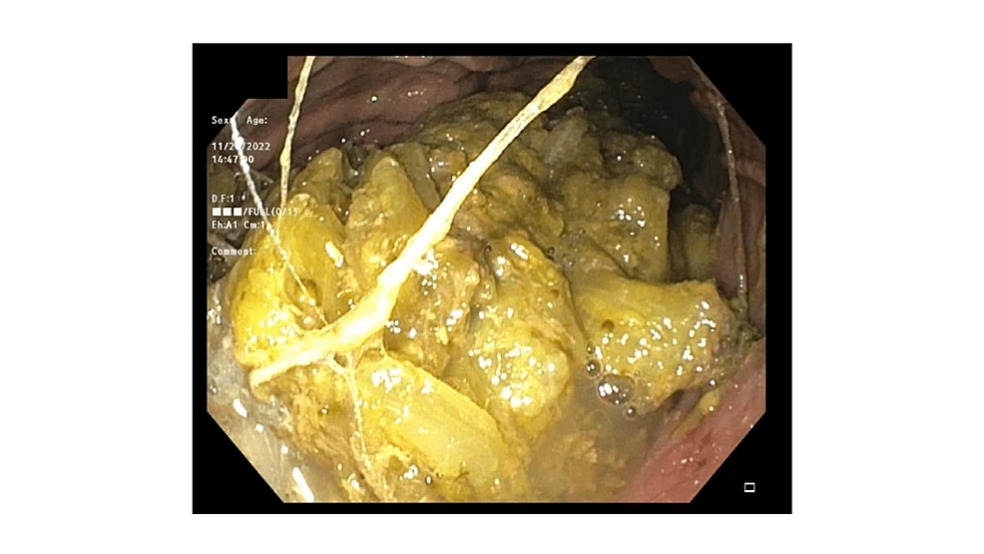
Solid food in stomach lumen on endoscopy

**Figure 2:**
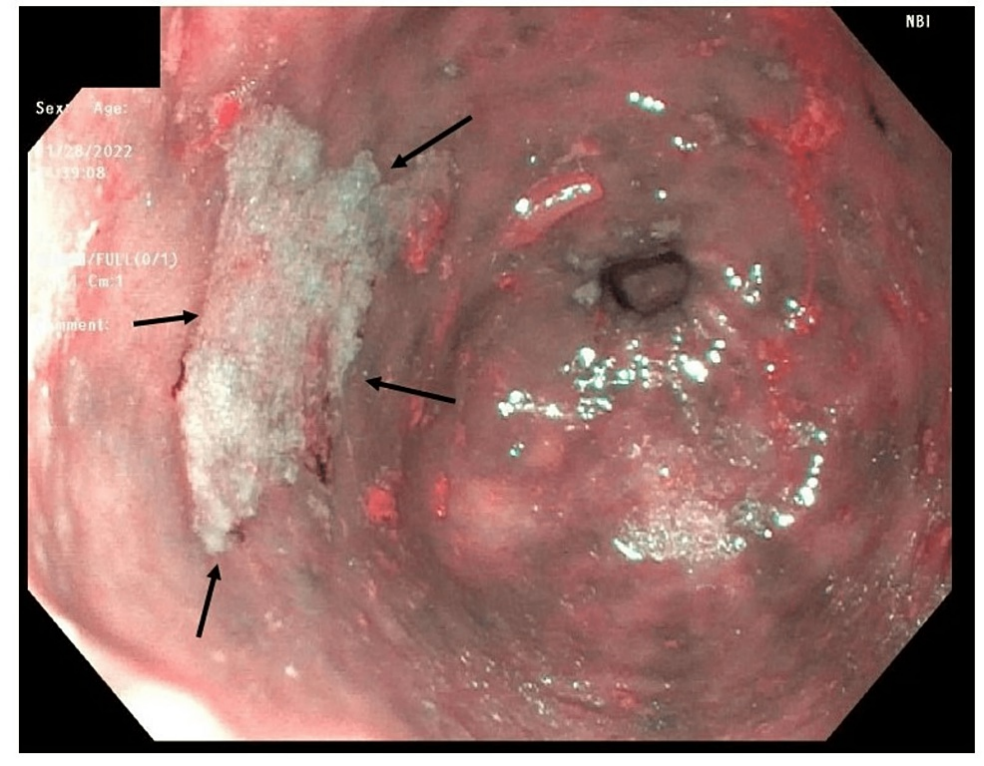
Discolored mucosa in gastric body Endoscopy revealed an area of pale discolored mucosa in the gastric body from which biopsies were obtained

The initial evaluation included a CT scan of the abdomen and pelvis, revealing circumferential thickening of the distal esophageal wall, mild gastric wall thickening possibly indicative of mild gastritis, and a significant stool burden in the colon. Subsequent upper endoscopy demonstrated a tortuous but normal distal esophagus without evidence of masses, strictures, or esophagitis. In response to the complaint of dysphagia, dilation of the gastroesophageal junction to 20 mm was performed without complications. Examination of the stomach revealed a substantial amount of solid food (Figure 1). Flattened, discolored, and atrophic mucosa displaying a diminished vascular pattern was noted in the gastric body (Figure 2).

**Figure 3:**
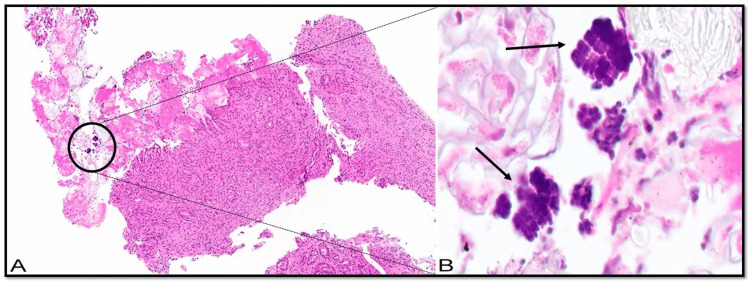
Histopathologic examination of stomach biopsies showing Sarcina organisms (A) Gastric mucosa with moderate to severe reactive gastropathy, ulceration, and acute fibrino-inflammatory exudate with embedded small tetrad organisms. The clusters are highlighted with a circle; (B) Higher magnification view showing large tetrads-shaped bacteria *Sarcina* organisms (Hematoxylin and eosin stain).

Biopsies were taken, and tissue pathology revealed moderate to severe reactive gastropathy, ulceration, and acute fibrino-inflammatory exudates (Figure 3A). Notably, large tetrad-shaped bacteria consistent with *Sarcina *spp. infection were identified (Figure 3B). The subsequent evaluation of the stomach and esophagus did not show signs of intestinal metaplasia or dysplasia.

Post-procedure, the patient was initiated on a seven-day course of oral ciprofloxacin (500 mg, twice daily), oral metronidazole (500 mg, three times daily), and a proton pump inhibitor for twelve weeks. Follow-up after two months showed a weight gain of 40 lbs. with diminished symptomatology. Repeat endoscopy with biopsy revealed the absence of *Sarcina *spp. infection or colonization, suggesting successful eradication and the resolution of atrophic mucosal plaques. However, new reactive esophagitis was observed during the follow-up endoscopy.

## Discussion

*Sarcina* spp. organisms are gram-positive aerotolerant anaerobic organisms recognized as zoo pathogens, often responsible for inducing "abdominal bloat" in bovines, primates, and pets [4,5,6]. The term zoo pathogens refers to pathogens, which are disease-causing microorganisms like bacteria, viruses, fungi, and parasites, that can be transmitted between animals in a zoo or wildlife setting. These pathogens can pose a significant risk to both the animals in captivity and the humans working with or visiting them. In humans, an association has been observed between gastrointestinal disorders involving delayed gastric emptying, such as diabetic gastroparesis, pyloric stenosis, and prior gastric surgeries with the potential for *Sarcina *spp. to become pathogenic [2,3]. The pathogenesis of *Sarcina *spp. infections is believed to involve mucosal injury in the context of reduced gastric motility, coupled with the bacteria's fermentative gas-producing metabolism [4].

While *Sarcina *spp. infections in humans remain infrequent, literature has seen an increase in reported cases over the past two decades, with approximately 70 reported cases indicating a potential rise in the incidence of this infection [3]. Several case reports have highlighted severe complications, including ulceration, gastric outlet obstruction, emphysematous gastritis, visceral perforation, and underlying malignancy, emphasizing the necessity for early detection of *Sarcina *spp. infections to prevent substantial morbidity and mortality [3,7-11]. Despite the growing number of severe complications associated with *Sarcina *spp., several cases have reported the presence of these organisms in the absence of symptoms or other pathological findings upon histologic review, suggesting a non-pathologic or opportunistic nature of these organisms [12-14].

In the presented case, the underlying gastroparesis likely contributed to the exacerbation of gastrointestinal symptoms and the pathogenesis of *Sarcina *spp., resulting in significant weight loss. The improvement of symptoms and weight gain following antimicrobial therapy supports the correlation between *Sarcina *spp. and the observed weight loss. However, it is essential to acknowledge that exacerbated dysphagia, dyspepsia, bloating/distention, and emesis may have also reduced oral intake, appetite, and nutritional absorption, contributing to significant weight loss.

Therapeutic approaches for *Sarcina *spp. infections are not yet established, but the most employed antimicrobial agent is metronidazole, alone or in combination with another antibiotic and high-dose proton pump inhibition [3]. The susceptibility profiles for *Sarcina *spp. organisms show efficacy of (fluoro)quinolones, macrolides, penicillins, and tetracyclines despite the type of host [6]. In the presented case, significant improvement in symptoms and substantial weight gain were observed following disease eradication. We propose consideration of *Sarcina *spp. infection as a complicating factor that should be treated when accompanied by deteriorating symptoms of reduced gastrointestinal motility, as a recent estimate of mortality based on reported cases was found to be 14% in patients, usually resulting in multi-organ failure from severe complications such as emphysematous gastritis, viscus perforation, peritonitis [3].

## Conclusions

This case exemplifies an uncommon cause of significant weight loss when accompanied by upper gastrointestinal symptoms, posing a potentially life-threatening risk if left untreated. Timely identification and management of *Sarcina *spp. infections in symptomatic patients are crucial in preventing significant morbidity and potentially life-threatening complications. Clinicians and pathologists should consider *Sarcina *spp. infection when presented with a case of unintentional weight loss and reduced gastric motility. Tissue biopsy should be considered in these cases as pathologic review is the preferred diagnostic tool in the evaluation to rule out malignancy and the identification of *Sarcina *spp. infection. Early intervention has the potential to mitigate undesired mortality outcomes.
